# Brachial artery access for endovascular procedures: how safe is it?

**DOI:** 10.1186/s42155-026-00741-4

**Published:** 2026-10-16

**Authors:** Lucy Rose Howroyd, Arjan Singh Khattar, Hoi-Ying Claudia Tam, Robert Morgan, Joo-Young Chun

**Affiliations:** Department of Interventional Radiology, St George’s University Hospital NHS Foundation Trust, London, SW17 0QT UK

**Keywords:** Brachial artery, Transbrachial, Endovascular, Access-site complications, Pseudoaneurysm, Vascular closure devices

## Abstract

**Purpose:**

While femoral and radial artery access are routine for endovascular procedures, brachial artery access remains an important alternative in selected cases. This study aimed to evaluate the complication rate of percutaneous brachial artery access at a tertiary centre and identify potential risk factors for access-site complications.

**Materials and methods:**

A retrospective review was performed of all procedures utilising percutaneous brachial artery access over a 12.5-year period. Patient demographics, sheath size, closure technique and complications were recorded. Complications were classified according to the CIRSE classification system.

**Results:**

One hundred percutaneous brachial artery punctures were identified. Sheath sizes ranged from 3 to 9 Fr; the majority (57%) using 6 Fr. Most procedures (84%) were performed for aortic, mesenteric/renal, or peripheral arterial interventions. Overall, 23 complications occurred (23%), including 9 (9%) grade 3 complications requiring additional treatment. No convincing association was identified between complication rate and patient age, gender, or sheath size. Vascular closure devices were used in seven cases, five of which resulted in complications, including three grade 3 events.

**Conclusions:**

The complication rate associated with brachial artery access in this series was higher than that reported in much of the published literature. These findings suggest that brachial artery access may be less safe than commonly perceived and that vascular closure devices should be used with caution, particularly given their off-label status for this access site. Standardised procedural protocols and post-procedural care pathways may help reduce complication rates. Broader auditing and reporting across centres are encouraged to better define best practice.

## Manuscript

### Introduction

Ultrasound-guided femoral and more recently radial artery access have become routine approaches for endovascular procedures. However, in selected clinical scenarios, brachial artery access remains a valuable alternative offering distinct technical advantages.

Upper-limb arterial access may be preferable to femoral access in patients with hostile groins, including those with severe peripheral arterial disease, prior surgical intervention, scarring, or infection. In certain anatomical configurations, brachial access can provide improved catheter stability and manoeuvrability, particularly when cannulating the mesenteric or renal arteries. Brachial artery access has been reported as favourable in carotid artery stenting [1] and as a useful adjunct to femoral access in complex endovascular aortic aneurysm repair [2].

Compared with femoral access, upper-limb approaches may also reduce time to ambulation, shorten recovery, and improve patient comfort [3–5], aligning with the increasing emphasis on day-case interventional radiology. Radial artery access, in particular, is well established in coronary angiography, demonstrating similar or reduced access-site complications and shorter hospitalisation compared with femoral access [6, 7].

However, radial artery access may be limited by vessel calibre, anatomical variation, contribution to palmar arch perfusion, and a maximum practical sheath size, often quoted as 7 Fr [8]. In addition, longer sheath lengths may reduce catheter manoeuvrability and device reach. In such cases, brachial artery access offers a larger calibre vessel and shorter working distance to target anatomy.

Despite these advantages, brachial artery access is generally associated with higher reported complication rates than femoral or radial access and is therefore less commonly used as a primary approach. Recognised complications include haematoma, thrombosis and occlusion, median nerve injury, pseudoaneurysm and arterial dissection. Published complication rates are often cited at approximately 11%, although reported rates vary widely, reflecting heterogeneity in complication definitions, access techniques, sheath sizes, and closure methods [9–20]. At our institution, brachial artery access is typically reserved for cases in which femoral or radial access are unsuitable or unsuccessful. We noted an increase in the number of patients who meet these criteria given the increasingly complex nature of our vascular patients. We also perceived a higher complication rate than suggested by much of the published literature.

The purpose of this study was to perform an updated evaluation of our institutional experience with percutaneous brachial artery access for endovascular procedures, with particular emphasis on determining the complication rate and identifying potential risk factors for access-site complications.

## Materials and methods

This was a single-centre retrospective study. Data were collected from the local radiology information system (RIS), picture archiving and communication system (PACS), and electronic patient records.

All interventional radiology procedures performed between 1 September 2013 and 30 May 2025 containing the terms “brachial artery” or “brachial access” in the procedure report were identified. Each case was reviewed, and only those in which percutaneous puncture of the brachial artery was confirmed were included. This resulted in a total of 100 cases.

For each case, patient age and gender, procedure type, sheath size, closure technique, and occurrence of complications were recorded. The gauge of puncture needle used, duration of manual compression, elbow immobilisation and post-procedural pressure dressing application was inconsistently documented and was recorded when available. Complications were classified according to the CIRSE Classification System [21]. Descriptive statistics were used for analysis due to sample size limitations.

Our current practice of brachial artery haemostasis is manual compression for at least 10 minutes, followed by further compression if haemostasis has not been achieved. The elbow is splinted with an arm board to minimise movement over the antecubital fossa for 4–6 hours and a duplex scan is usually performed prior to discharge.

## Results

One hundred percutaneous brachial artery punctures were performed during the study period by nine operators (all brachial punctures and closures were performed under direct or indirect supervision by a consultant interventional radiologist. The distribution of procedure types is shown in Table 1. All access was obtained using ultrasound-guided percutaneous puncture of the brachial artery in the antecubital fossa.

**Table 1:** Types of procedures where brachial artery access used

Vascular intervention type	Total (*n* = 100)
Aortic/mesenteric/renal arterial	41
Carotid/subclavian/upper limb arterial	29
Dialysis AV fistula	15
Iliac/lower limb arterial	12
Axillary-femoral arterial bypass reintervention	2
Goitre embolisation	1

There were 64 male and 36 female patients, with a mean age of 69 years. Sheath sizes ranged from 3 to 9 Fr; 57% of cases utilised a 6 Fr sheath. Twelve cases used a sheath larger than 6 Fr. Sheath size was not documented in nine cases without complications. Low profile radial sheaths were not used. Sheath length is not routinely recorded as part of our procedure protocol. Seven cases utilised a vascular closure device rather than manual compression.

A total of 23 complications occurred, yielding an overall complication rate of 23%. Fourteen complications were classified as CIRSE grade 1 or 2, requiring prolonged compression or observation without additional therapy. Nine complications (9%) were classified as CIRSE grade 3 (Table 2). Five pseudoaneurysms were treated successfully with a single thrombin injection during admission; one pseudoaneurysm was treated unsuccessfully with thrombin and required surgical closure. In one case, there was prolonged bleeding and an expanding haematoma, and the patient was taken to theatre for surgical closure. In one case, a large haematoma formed, and the patient required blood products and additional follow-up. Finally, there was one symptomatic arterial occlusion that required unplanned anticoagulation and prolonged monitoring. All but one complication was diagnosed during the same admission; one asymptomatic occlusion was noticed some months later when the patient returned for a repeat procedure.

**Table 2:** Complications relating to brachial artery access

Complication	Grade 1 or 2 (*n* = 14)	Grade 3 (*n* = 9)
Haematoma/prolonged bleeding	11	2
Pseudoaneurysm	0	6
Dissection	1	0
Occlusion	2	1

Table 3 summarises the risk factors for complication analysed. In cases without complications (*n* = 77), sheath sizes ranged from 3 to 9 Fr (median 6 Fr). In cases with complications (*n* = 23), sheath sizes ranged from 4 to 6 Fr (median 6 Fr). In grade 3 complications (*n* = 9), sheath sizes ranged from 4 to 7 Fr (median 6 Fr).

**Table 3:** Potential complication risk factor analysis

Variable	No complication (*n* = 77)	Complication (*n* = 23)	*p*-value
Female sex, *n* (%)	26 (33.8)	10 (43.5)	0.461
Age, median (years)	69	70	0.178*
Sheath size > 6 Fr, *n* (%)	10 (14.7)	3 (13.0)	1.000
Closure device,* n* (%)	2 (2.6)	5 (21.7)	0.0067

*Mann–Whitney *U* test. All other *p*-values from Fisher’s exact test

The mean age was 68 years in patients without complications and 71 years in those with complications. Patients with grade 3 complications had a mean age of 76 years. Female patients accounted for 26/77 cases without complications and 10/23 cases with complications; among grade 3 complications, four occurred in female patients.

Regarding closure technique, most cases were managed with manual compression, with or without a compression bandage and arm splint. Closure devices were used in seven cases overall—Angio-Seal ™ VIP (Terumo Medical, New Jersey, USA) and MYNX CONTROL™ (Cordis, Florida, USA). The reason for using a closure device was not specified in any report. Five of these seven cases (71%) were associated with complications, including three grade 3 events (two pseudoaneurysms requiring thrombin injection and one surgical repair). The details of the cases where a closure device was used are summarised in Table 4.

**Table 4:** Details of cases in which a vascular closure device was used

Age	Gender	Sheath size	Type of case	Closure device	Complication	CIRSE grade of complication
63	F	6	Dialysis AV fistula	Angio-Seal ™ VIP	Prolonged bleeding	1
87	F	6	Mesenteric	MYNX CONTROL™	Pseudoaneurysm	3
69	F	6	Dialysis AV fistula	Angio-Seal ™ VIP	Focal occlusion	1
46	F	7	Lower limb	Angio-Seal ™ VIP	Symptomatic occlusion	3
81	F	6	Mesenteric	Angio-Seal ™ VIP		0
52	M	7	Lower limb	Angio-Seal ™ VIP		0
91	M	6	Subclavian	Angio-Seal ™ VIP	Pseudoaneurysm	3

Secondary analysis of the grade 3 complications (*n* = 9) demonstrated no significant associated with gender or sheath size. There was a trend towards increasing age amongst patients experiencing grade 3 complications (median 78 vs 68 years, *p* = 0.055). Closure device use was associated with grade 3 complications (42.9% vs 6.5%, Fisher’s exact *p* = 0.03), although only seven closure devices were used and this finding should therefore be interpreted with caution.

## Discussion

This study demonstrates an overall complication rate of 23% associated with percutaneous brachial artery access, with 9% classified as CIRSE grade 3 complications. This rate is higher than that reported in many published series, although substantial heterogeneity exists within the literature.

Previous studies have identified age, female gender, and larger sheath size as risk factors for brachial artery access complications [5, 22–24]. In the present study, no convincing association was observed between complication rate and age or sheath size. Female gender appeared more common among patients experiencing grade 3 complications, although the limited sample size precluded robust statistical analysis. This finding is consistent with prior work by Alvarez-Tostado et al., who reported female gender as the only significant demographic risk factor [13]. Hypertension has also been identified as an independent risk factor for brachial artery pseudoaneurysm formation [23]; however, retrospective documentation limitations prevented reliable assessment of peri-procedural blood pressure in this cohort. Use of antiplatelet or anticoagulation may also be a risk factor for complications, although we were unable to capture this information reliably in our cohort, patients with peripheral arterial disease are routinely on single or dual antiplatelet therapy at the time of the procedure. On the other hand, anticoagulation is discontinued with a target INR < 1.5 or DOAC discontinued for 48 h prior to the procedure.

Notably, no cases of median nerve injury were observed, a recognised but rare complication of brachial artery access [25, 26]. We attribute this, at least in part, to routine ultrasound guidance and operator familiarity with upper-limb neurovascular anatomy. Ultrasound guidance is strongly advocated to minimise access-related complications and should be considered standard practice.

The use of vascular closure devices in the brachial artery remains controversial and is off-label. Although recent meta-analyses have suggested high technical success rates without increased complications [27], our experience differed. Although limited by small numbers, complications occurred in five of seven cases in which closure devices were used, including three grade 3 events. These findings suggest that closure devices should be used with caution in the brachial artery, and operators should remain mindful of their off-label status.

Considerable variability was observed in closure techniques and post-procedural care instructions, reflecting both operator preference and the absence of clear evidence-based guidance. Additionally, the rationale for selecting brachial access over femoral or radial access was not consistently documented. Given the potentially higher complication rate associated with brachial artery access, explicit documentation of access selection rationale may be beneficial.

The observed complication rate may also reflect the case mix at a tertiary referral centre, where patients frequently present with complex anatomy or limited alternative access options. The retrospective nature of the study limits control over confounding factors, including anticoagulation status, operator experience, puncture level along the brachial artery, and peri-procedural haemodynamics.

Importantly, this work directly informed departmental practice, leading to the implementation of a standard operating procedure (SOP) and structured reporting template to standardise access technique, closure, documentation, and follow-up. Patients are counselled appropriately during informed consent when brachial artery access may be required. We have reiterated the need for careful technique during brachial artery puncture. Ultrasound-guided careful single wall puncture is recommended, avoiding double wall or multiple punctures. Additionally, care should be taken to ensure atraumatic insertion of the sheath and extra attention devoted to the removal of the sheath and manual pressure. Finally, we have reiterated in the department that haemostasis is a key part of the overall procedure and should not be left to an unsupervised junior trainee. These measures aim to improve patient safety and facilitate future re-audit.

## Conclusions

Percutaneous brachial artery access was associated with a higher complication rate at our institution than that reported in much of the published literature. These findings suggest that brachial artery access may be less safe than commonly perceived and should be reserved for carefully selected cases. Vascular closure devices should be used with caution given their off-label status and observed complication profile. Standardised procedural protocols and post-procedural care pathways may help reduce complications. Wider auditing and transparent reporting across centres are needed to better define best practice and improve patient outcomes.

## Data Availability

Data can be acquired upon reasonable request to the corresponding author.

## Abbreviations

CIRSE: Cardiovascular and Interventional Radiological Society of Europe
RIS: Radiology information system
PACS: Picture archiving and communication system
SOP: Standard operating procedure
